# Optimization and Bioactive Evaluation of *Bifurcaria bifurcata* Antioxidant-Rich Extracts for Functional Food and Pharmaceutical Applications

**DOI:** 10.3390/antiox13101189

**Published:** 2024-09-30

**Authors:** Aurora Silva, Maria Carpena, Lucia Cassani, Clara Grosso, Paula Garcia-Oliveira, Cristina Delerue-Matos, Jesus Simal-Gandara, Maria Fatima Barroso, Miguel A. Prieto

**Affiliations:** 1 Universidade de Vigo, Nutrition and Bromatology Group, Department of Analytical Chemistry and Food Science, Instituto de Agroecoloxía e Alimentación (IAA)—CITEXVI, 36310 Vigo, Spain; mass@isep.ipp.pt (A.S.); mcarpena@uvigo.es (M.C.); paula.garcia.oliveira@uvigo.es (P.G.-O.); jsimal@uvigo.es (J.S.-G.); 2 REQUIMTE/LAQV, Instituto Superior de Engenharia do Porto, Instituto Politécnico do Porto, Rua Dr. António Bernardino de Almeida 431, 4249-015 Porto, Portugal; claragrosso@graq.isep.ipp.pt (C.G.); cmm@isep.ipp.pt (C.D.-M.)

**Keywords:** microwave-assisted extraction, nutraceuticals, bioactive properties

## Abstract

In recent years, consumers have been increasingly interested in natural, healthier, functional foods, with a focus on sea-based products such as algae. *Bifurcaria bifurcata* (BB) is a macroalga that belongs to the Phaeophyceae class. These brown algae are recognized as the source of bioactive molecules of great interest to the pharmaceutical and nutraceutical industries. The present work applied response surface methodology to optimize the microwave-assisted extraction of the poorly studied algae. The optimization variables were time, pressure, and solvent composition (ethanol/water) and the response parameters selected were yield, total phenolic and flavonoid content, and the antioxidant profile by evaluating DPPH^•+^, ABTS^•+^ scavenging activity, and β-carotene discoloration capacity. The results obtained reveal remarkable bioactivity of the crude extract of BB with positive results as an antioxidant and antimicrobial agent. Furthermore, the BB extract’s capacity to inhibit enzymes related to neurodegenerative diseases and its anti-inflammatory and anti-proliferation activity open the possibility of future food or pharmaceutical applications.

## 1. Introduction

Recently, there has been a noticeable interest in sea-based products, especially seaweeds, due to their richness in bioactive molecules [1,2]. Several studies have reported the presence of bioactive molecules with special emphasis on brown algae [3,4]. *Bifurcaria bifurcata* (BB) R. Ross is a macroalga belonging to the Phaeophyceae class, mostly found in naturally occurring ponds distributed on the Northwest Atlantic coast during low and medium tides [5,6]. This species is an edible alga, rich in dietary-fiber-containing uronic acids from alginates and neutral sugars [7]. The specialized literature reports that BB has in its composition biomolecules such as polyphenols, pigments, and fatty acids and is especially rich in linear diterpenes [8,9]. Furthermore, its extracts obtained with different solvents (acetone, hexane, ethanol, water, etc.) have been shown to have antioxidant [10,11], antitumoral [12], and antimicrobial activities [13].

There are a considerable number of extraction techniques that have been applied to seaweeds seeking the recovery of bioactive molecules; however, the most frequent method used to extract and recover these high-value bioactive compounds is maceration [6]. Nonetheless, it is crucial to develop and apply more highly efficient, eco-friendly extraction methods.

Microwave-assisted extraction (MAE) presents a substantial number of benefits over traditional solid–liquid techniques, and its application allows a more environmentally friendly process due to the lower quantity of solvents used and the higher extraction rates achieved. MAE has been successfully applied to various matrices, particularly natural products [14]. This technique, when applied to macroalgae [15,16,17,18], proves to be effective in the recovery of bioactive molecules, namely, polyphenols from brown algae, when compared with traditional extraction techniques [4]. However, MAE efficiency is heavily dependent on the operating conditions and parameters impacting the extraction mechanisms and yield. Some of those critical parameters are the extraction time, the nature of the solvent used, and the temperature/pressure utilized [19].

For the MAE extraction optimization, a traditional approach (to keep constant all parameters except the one under study) is arduous and time-consuming and does not allow the researchers to infer the interaction between variables. Hence, the response surface methodology allows the optimization of responses as a function of multiple variables at the same time, consequently minimizing the number of experimental runs required [20]. In this work, a circumscribed central composite design (CCCD) was used to study the influence of the operational variables of MAE. This mathematical approach has already proven its advantages in the optimization of bioactive compound extraction from different natural matrixes such as chestnut flowers and algae, among others [21].

Based on previous studies, the MAE operation parameters chosen to optimize were time, pressure, and solvent composition as independent variables to maximize total phenolic content (TPC), and the antioxidant profile: 2,2-diphenyl-1-picrylhydrazyl radical scavenging activity (DPPH^•^), 2,2′-azinobis(3-ethylbenzothiazoline-6-sulfonic acid (ABTS^•+^) scavenging activity, and β-carotene bleaching (BCM) [22].

Once the optimal extraction conditions were established, the extract obtained was evaluated in terms of its potential bioactivity. For that, the selection of parameters was made from four different perspectives: food spoilage prevention, oxidative stress as a scavenger of nitrogen and oxygen reactive species, neuroprotective capacity, and anti-inflammatory and cytotoxic potential.

The antioxidant and antimicrobial capacities are very important in food preservation since they are two major factors in food spoilage [23]. The following foodborne microorganisms were selected: *Staphylococcus aureus*, *Bacillus cereus*, *Pseudomonas aeruginosa*, *Salmonella enteritidis*, and *Escherichia coli*, and additionally *Staphylococcus epidermidis*, an opportunistic skin bacterium, was also studied [24].

Based on published work reporting the activity of these algae on brain disorders [10], we studied the neuroprotective action through the inhibition of key enzymes involved in the pathogenesis of neurodegenerative and neuropsychiatric disorders, such as cholinesterases (related to Alzheimer’s disease), and tyrosinase and monoamine oxidase (MAO) A and B (related to Parkinson’s disease and depression) [25]. Furthermore, the scavenging capacity of reactive oxygen and nitrogen species was also studied since oxidative and nitrosative stresses are important triggers of neuroinflammation [26].

The potential as a cytotoxic and anti-inflammatory agent is supported by previous work on BB active compounds [9,27]; for that, cell-based assays were conducted on the anti-inflammatory capacity, hepatotoxicity (Vero), and the cytotoxicity to cell lines of lung adenocarcinoma (A549), gastric adenocarcinoma (AGS), and hepatocellular carcinoma (HepG2).

## 2. Materials and Methods

### 2.1. Material and Reagents

Except for ethanol, supplied by Carlo Erba Reagents S.A. (Emmendingen, Germany), hydrogen peroxide, purchased from Laborspirit (Lisboa, Portugal), and salicylic acid, which came from Fisher Scientific (Leicestershire, UK), all the chemicals were purchased from Sigma-Aldrich (St. Louis, MO, USA, and Steinheim, Germany). Microbiologics, MN (St. Cloud, MN, USA) supplied *E. coli* (NCTC 9001) and *S. epidermidis* (NCTC 11047); Selectrol, (Buckingham, UK), supplied *S. aureus* (ATCC 25923), *B. cereus* (ATCC 14579), *P. aeruginosa* (ATCC 10145), and *S. enteritidis* (ATCC 13076).

### 2.2. Algae Collection and Preparation

BB was handpicked (19 specimens) on the northwestern Galician coasts by the Algamar company (www.algamar.com, accessed on 10 July 2024) and provided for this work. The BB was received fresh and washed with deionized water to remove superficial debris. The algae were conserved at −80 °C, lyophilized (LyoAlfa 10/15 from Telstar, Shanghai, China), and reduced to powder (~20 mesh). This powder was blended to ensure samples were representative and stored (−20 °C) till use.

### 2.3. Microwave-Assisted Extraction

The extractions were carried out using a Multiwave-3000 microwave reaction system (Anton Paar, Ostfildern-Scharnhausen, Germany). Briefly, in each microwave vessel, 0.6 g of powder algae was mixed with 20 mL of extraction solvent. A 1400 W irradiation power was applied, and magnetic stirring was maintained throughout the process. Immediately after the extraction, the vessels were cooled (ice bath, 5 min), transferred to falcons and centrifuged (8400 rpm, 15 min), and stored at −80 °C [22].

### 2.4. Experimental Design, Modeling, and Optimization

The MAE extraction was optimized aiming to obtain an antioxidant-rich extract from BB. For that, the critical conditions time (t), pressure (P), and solvent composition (ethanol/water) (S), were studied using a factorial experiments approach applying a circumscribed central composite design (CCCD) [28]. Subsequently, a CCCD of five levels was employed to investigate the impacts on five dependent variables, resulting in a total of 28 response permutations, to improve the model’s predictive ability. The codification of independent variables is presented in Table 1.

### 2.5. Model Independent Variables

The effects of time (t), pressure (P), and ethanol concentration (S) on BB extraction were investigated at the same time using extraction yield, total phenolic content (TPC), and antioxidant activity through DPPH^•^ and ABTS^•+^ radical scavenging activity and β-carotene bleaching assays as response. Experimental protocols are briefly described below.

#### 2.5.1. Yield

To calculate the extraction yield, the dry weight of the crude extract (E) (dry 105 °C until constant weight) was divided by the mass of lyophilized algae utilized in each extraction point (mg E/g) [22].

#### 2.5.2. Total Polyphenol Content (TPC)

TPC was determined by the Folin–Ciocalteu method. Briefly, 24 μL of each extract was added to 120 μL of the Folin–Ciocalteu reagent (1:10 *v*:*v*). After 3 min of incubation (RT), 96 μL of Na_2_CO_3_ solution (7.5% *w*/*v*) was added and the reactive mixture was incubated for 2 h. The absorbance was measured at 765 nm [29]. The results were expressed in mg of phloroglucinol equivalents (FhL)/gE (dw).

#### 2.5.3. DPPH^•^ Scavenging Activity

The antioxidant capacity was studied based on the DPPH^•^ radical scavenging method referenced before [30]. A methanolic DPPH solution (76 mM) was properly diluted to give absorbances between 1.2 and 1.3. After this, 40 µL of each extract and 200 μL of the DPPH reagent were added to each well. The reactive mixture was incubated at RT in the dark for 60 min and absorbance was measured at 515 nm. Results were expressed in nM DPPH^·^/g E (dw).

**Table 1:** The CCCD’s experimental RSM results for the MAE optimization of the independent variables’ (*X*_1_, *X*_2_, and *X*_3_) assessed responses (Yield, TPC, DPPH, ABTS, and BCM). Variables are presented in natural values and codified ranges.

	Experimental Design						Responses				
	Coded Values			Natural Values							
	*X*_1_	*X*_2_	*X*_3_	*X*_1_ *: t*	*X*_2_ *: P*	*X*_3_ *:* *S*	*Y*	*TPC*	*DPPH* ^•^	*ABTS*	*BCM*
				min	Bar	%	mg/g dw	mg FE/g dw	nM R·/g dw	nM R·/g dw	µM βC/g dw
1	−1	−1	−1	7.5	5.6	20.3	426.77	57.12	32.70	41.73	0.0902
2	−1	−1	1	7.5	5.6	79.7	330.76	46.50	32.12	159.51	0.1037
3	−1	1	−1	7.5	16.4	20.3	514.74	69.46	39.99	44.61	0.1060
4	−1	1	1	7.5	16.4	79.7	362.82	52.28	34.79	280.31	0.0354
5	1	−1	−1	20.5	5.6	20.3	541.44	61.89	42.05	53.86	0.1279
6	1	−1	1	20.5	5.6	79.7	370.05	52.19	29.83	56.22	0.0481
7	1	1	−1	20.5	16.4	20.3	462.27	61.70	41.84	29.30	0.0913
8	1	1	1	20.5	16.4	79.7	326.94	48.42	26.19	184.17	0.0712
9	1.68	0	0	25	11	50	423.36	58.72	32.49	168.26	0.0806
10	−1.68	0	0	3	11	50	339.81	64.29	44.94	165.00	0.0870
11	0	−1.68	0	14	2	50	323.71	44.04	30.59	84.13	0.0950
12	0	1.68	0	14	20	50	457.90	63.23	38.15	85.67	0.0757
13	0	0	−1.68	14	11	0	452.43	43.58	26.07	180.96	0.0959
14	0	0	1.68	14	11	100	100.33	19.23	13.25	22.51	0.0017
15	−1.68	−1.68	−1.68	3	2	0	283.57	24.48	12.20	216.08	0.0677
16	−1.68	−1.68	1.68	3	2	100	59.64	6.07	2.22	4.46	0.0017
17	−1.68	1.68	−1.68	3	20	0	393.25	33.17	17.52	18.40	0.0781
18	−1.68	1.68	1.68	3	20	100	115.62	16.39	11.76	19.92	0.0046
19	1.68	−1.68	−1.68	25	2	0	345.64	31.39	16.46	181.43	0.1029
20	1.68	−1.68	1.68	25	2	100	55.48	2.93	1.46	2.70	0.0017
21	1.68	1.68	−1.68	25	20	0	365.07	32.35	17.77	36.29	0.0391
22	1.68	1.68	1.68	25	20	100	112.57	11.38	14.17	36.33	0.0096
23	0	0	0	14	11	50	385.65	62.49	33.79	169.41	0.0946
24	0	0	0	14	11	50	371.11	69.10	32.96	195.22	0.0950
25	0	0	0	14	11	50	411.94	62.68	27.71	199.82	0.0908
26	0	0	0	14	11	50	369.34	62.99	30.63	119.88	0.0926
27	0	0	0	14	11	50	400.93	59.00	23.27	180.36	0.1029
28	0	0	0	14	11	50	380.33	48.41	23.03	183.10	0.0959

#### 2.5.4. Azino-Bis(3-Ethylbenzothiazoline-6-Sulfonic Acid) ABTS^•+^ Scavenging Activity

The technique followed the previously proposed methodology [22], and the radical ABTS^•+^ was generated by the interaction of 19.3 mg of dissolved ABTS in 5 mL of water and 88 μL of potassium persulfate (K_2_S_2_O_8_) (0.0378 g/mL). The mixture was incubated in the dark at RT for 16 h. After this, 250 μL of the ABTS^•+^ radical was mixed with 10 mL of ethanol until an absorbance between 1.3–1.4 units was measured at 734 nm. The reaction started with 200 μL of the ABTS^•+^ radical solution previously prepared and 40 μL of each extract. After a 60 min incubation at RT, absorbance was measured at 734 nm. The results were expressed as nM ABTS^•+^/g E (dw).

#### 2.5.5. β-Carotene Discoloration Method (BCM)

To study the *β-carotene* (*βC*) discoloration action. Succinctly, a mother β-carotene solution was prepared at 1 mg/mL in chloroform and, after 1 mL was added, was mixed with 20 mL of polysorbate (surfactant) and 10 mL of chloroform in a flask. After complete dissolution, the solvent was evaporated in a rotavapor operating at 40 °C and redissolved in 300 mL of ultrapure water. Finally, 0.5 mL of linoleic acid was added to the aqueous solution of β-carotene and stirred vigorously. The reaction started with 200 μL of the β-carotene solution and 40 μL of each extract, and the absorbance at 470 nm was determined after incubation at 45 °C for 60 and 120 min. Results were expressed as µM βC/g E (dw) [31].

All the above microplate methods were performed in triplicate and conducted in a Synergy ™ HTX microplate reader (BioTek Instruments, Winooski, VT, USA).

### 2.6. Mathematical Model

A minimal squares regression was applied to a third-order polynomial equation with interactive terms of Equation (1) to fit the response surface methodology (RSM) data.

(1) $$Y=b_{0}+\sum_{i=1}^{n}b_{i}X_{i}+\sum_{\begin{array}{c} i=1\\ j>i \end{array}}^{n-1}\sum_{j=2}^{n}b_{ij}X_{i}X_{j}+\sum_{i=1}^{n}b_{ii}X_{i}^{2}+\sum_{\begin{array}{c} i=1\\ j>i \end{array}}^{n-1}\sum_{j=2}^{n}b_{iijj}X_{i}^{2}X_{j}^{2}+\sum_{\begin{array}{c} i=1\\ j>i \end{array}}^{n-2}\sum_{j=2}^{n-1}\sum_{\begin{array}{c} k=3\\ k>j \end{array}}^{n}b_{ijz}X_{i}^{2}X_{j}^{2}X_{k}^{2}+\sum_{i=1}^{n}b_{iii}X_{i}^{3}$$

*Y* represents the mathematical expression of the dependent variable (response) to be modelled, namely, Yield, TPC, DPPH^•^, ABTS^•+^, or BCM. *X_i_* and *X_j_* are the independent variables *t*, *P*, and *S*, *b*_0_ is a constant coefficient, *b_i_* describes the linear individual contribution of each variable, *b_ij_* is the coefficient that defines the interaction between the several variables, and *b_ii_* is the coefficient related to the quadratic effect of each variable. The coefficients *b_iijj_* and *b_iii_* represent the quadratic interactive mechanisms between two variables, and the cubic effect of each variable n stands for the number of variables. The interactive quadratic and cubic terms allow the predictive model to fit properly by representing the complex interactions between the three independent variables. Function limits were imposed on the coded values to prevent unnatural or odd results namely *t* > 0 and 0 > *S* ≥ 100.

### 2.7. Numerical Methods, Statistical Analysis, and Figures

A simplex method to solve non-linear problems was applied to optimize the model and maximize the response in terms of experimental responses (yield, TPC, and parametric values of the antioxidant methods (ABTS^•+^, DPPH^•^, and βC)). For that, the quasi-Newton algorithm (least-square) was run conducing to the parameter’s adjustment by minimizing the quadratic differences between observed and predicted values. The ‘SolverAid’ macro in Microsoft Excel (Office 365) was used to evaluate the parametric confidence intervals and the coefficients significance α = 0.05. Finally, the model robustness was assessed by Fisher F-test (α = 0.05), and the model estimate uncertainties were analyzed through the ‘SolverStat’ macro [32], the *R*^2^ identified the percentage of variability in the dependent variable, and the Durbin–Watson coefficient (DW) was used to assess the autocorrelation of the model’s residuals.

The IC_50_ values were estimated by fitting the Weibull model [33] (Equation (2)). This mathematical approach has already been shown to be a robust methodology to accurately represent dose–response systems [22]. The distribution equation can be presented as follows:

(2) $$Y\left(X\right)=K[1-EXP\;(-Ln2{\left(\frac{X}{{IC}_{50}}\right)}^{a})]$$

The adjusted parameters were the dose–response curve slope (a) and asymptote (K), and the IC_50_ value. These parameters were calculated with a 95% confidence level and presented a normal distribution of residues *p* < 0.05 (Shapiro–Wilk test). The calculations were performed with the GraphPad prism 8 software. Three-dimensional graphic illustrations were created using the Deltagraph 5 software.

### 2.8. Biological Activities Assessment at the Optimal BB Extract

#### 2.8.1. Antimicrobial Assay

BB extract was dissolved in dimethyl sulfoxide (DMSO) to a final concentration of 20 mg/mL and sterilized by filtration (0.20 µm syringe filter, CHM Barcelona, Spain). All microorganisms were cultivated in Mueller–Hinton broth (MHB) overnight at 37 °C as the pre-inoculum, and after that the bacteria concentration was standardized to 0.5 MacFarland scale corresponding to 0.1 ± 0.01 absorbance measured at 600 nm [29].

Antimicrobial activity was assessed by plate diffusion method and 50 µL of the microorganism to be tested was seeded into Petri dishes containing Mueller–Hinton agar (MHA). Subsequently, 15 µL of extract, 15 µL of DMSO (negative control), and 15 µL of 40% (*v*/*v*) lactic acid (positive control) were added. Using a digital caliper, the inhibition zone diameter was determined after 24 h of incubation at 37 °C [29,34]. The minimal inhibitory concentration (MIC) was performed by the microdilution method [29]. Briefly, a 96-well round bottom sterile plate was filled with a total volume of 250 µL, containing 10^6^ CFUs, algae extract (100 µL), and fresh MHB media. Sample blanks of non-inoculated medium with extracts, positive controls prepared with inoculated medium, and negative control lactic acid (40%) were included in each test. The experiments were conducted for 24 h at 37 °C at 630 nm.

#### 2.8.2. ROS and RNS Scavenging Activity

BB antiradical activity towards the superoxide (O_2_^●−^) was studied by the reaction between phenazine methosulfate (PMS) and nicotinamide adenine dinucleotide (NADH) which generates the radical O_2_^●−^ and then reacts with nitroblue tetrazolium (NBT). Extracts were dissolved in KH_2_PO_4_/K_2_HPO_4_ buffer (19 mM; pH 7.4) and six serial dilutions were prepared. Subsequently, 50 μL of extract (from 0.065 to 2 mg/L), 50 μL of NADH 166 μM, 150 μL of NBT 43 μM, and finally 50 μL of PMS 2,7 μM were added. Kinetic curves were obtained at 560 nm [35,36].

^●^NO scavenging activity was evaluated by a methodology based on a diazotization reaction. Briefly, six different concentrations of BB extract (phosphate buffer 0.1 M pH = 7.4) were incubated with sodium nitroprusside 20 mM for one hour at RT under natural illumination. After this time, Griess reagent (1% sulphanilamide + 0.1% naphthylethylenediamine in 2% phosphoric acid) was added and the mixture was set to rest for 10 min. The plate was read at 560 nm, phosphate buffer was used as negative control, and 2% phosphoric acid, instead of the Griess reagent, was added to the blanks [35].

H_2_O_2_ scavenging ability was carried out based on the decreasing of the signal at 230 nm. For that, 425 µL of each of the 6 tested extract concentrations (dissolved in 0.1 M phosphate buffer pH = 7.4) was added to 75 µL of 40 mM H_2_O_2_ solution. After 10 min, the absorbances were read in a Shimadzu UV-260 spectrophotometer (Kyoto, Japan). A blank sample was made for each dilution by replacing the 75 µL of H_2_O_2_ solution with buffer, and buffer solution was used as the negative control [37,38].

The scavenging of radical hydroxyl (^●^OH) was carried out based on the salicylic acid method as described previously [39]. Extract samples were diluted in water and six serial dilutions were prepared. After that, 70 µL of each extract concentration, 70 µL of 9 mM salicylic acid, 70 µL of 9 mM iron sulfate, and 70 µL of H_2_O_2_ 9 mM were added. The microplate was incubated at 37 °C for 60 min and absorbance was read at 510 nm. Sample blanks and negative controls were made in all experiments. Ascorbic acid was used as the reference for all assays. The micro methods were carried out in a Synergy ^HT^ (BioTek Instruments, Winooski, VT, USA) microplate reader. All the experiments were performed in triplicate (n = 3) and results were expressed as IC_50_ µg/mL.

#### 2.8.3. Enzyme Inhibition Assays

Cholinesterase (AChE and BuChE) inhibition capacity was performed by Ellman’s method based on the measurement of the thiocholine released during the acethylocholine hydrolysis under the influence of AChE or BuChE. In each well, 25 µL of extract, 125 µL of 3 mM DTNB, 25 µL of substrate (ATCI or BTCI), and finally 50 µL of buffer (Tris-HCl; pH = 8) was added; the reaction kinetics were monitored at 405 nm for 2 min. Galantamine was used as a positive control [35].

Monoamine oxidase A (MAO–A) and B (MAO–B) inhibition activity caused by BB extracts was evaluated by measuring the production of 4-hydroxyquinoline at 314 nm for 70 min, using kynuramine 3.75 mM as substrate, according to previously published work [35]. Extracts were dissolved in 0.1 M potassium phosphate buffer pH 7.4. The reaction was initiated by adding 75 µL of 17 U/mL MAO–A or MAO–B solution. Clorgyline was used as a positive control.

Tyrosinase inhibition was assessed as described by Masuda et al. [40]. BB extract was dissolved in phosphate buffer (1/15 mM, pH = 6.8) and six serial dilutions were prepared. To each well, 40 µL of sample or buffer (negative controls), 80 µL of buffer, and 40 µL of tyrosinase 46 U/mL were added and incubated for 10 min at RT. After that, 40 µL of L-DOPA 2.5 mM was added and incubated for 10 min more. Finally, the absorbance was read at 475 nm. Kojic acid was used as a positive control.

Buffer replaced extracts (in the case of negative controls) and enzymes or substrates (for blanks). All tests were performed in triplicate and results were presented as IC_50_ (µg/mL).

#### 2.8.4. Cell Line Studies

##### Anti-Inflammatory Activity

Pre-incubated (24 h, 37 °C, 5%CO_2_; 5 × 10^5^ cells/mL) RAW264.7 murine macrophages were transferred to a microplate containing BB extracts in DMEM (Dulbecco’s Modified Eagle’s Medium) and incubated for 1 h before adding LPS (Lipopolysaccharide) to induce an inflammatory response. After 24 h, the supernatant was transferred to another microplate and the NO level was evaluated by the Griess reagent method [41].

##### Cytotoxicity Activity Assay

The sulforhodamine B (SRB) methodology was used to assess the cytotoxic activity of the BB crude extracts. A microplate was prepared with 10 µL of BB (8 mg/mL four dilutions) and, supplemented with 10 µL of HBSS and 190 µL suspension of 5 × 10^4^ cells/mL, was incubated for 48 h at 37 °C, 5% CO_2_ conditions. After that, the cells were fixed with 10% TCA (trichloroacetic acid) and dyed with 100 µL SBR (30 min room temperature). The microplate was carefully washed (1% acetic acid) and the protein-bound dye dissolved in Tris (10 mM), and finally the absorbance was measured at 540 nm [42].

## 3. Results and Discussion

### 3.1. Mathematical Modelling of the Optimization

Table 1 presents the average values (n = 3) of the tested independent variables for each of the 28 conditions (*t*, *P*, and *S*) used in the RSM optimization. Equation (1) was used to fit the experimental data to the polynomial models and construct the 3D surface graphical responses. Table 2 displays the calculated regression coefficients with statistical significance (*p* < 0.05) resulting from the model fit analysis.

The coefficients found to be non-significant (ns) were not considered in the development of the model and are not shown. The reduced versions of the polynomial equations for each of the responses examined are as follows, using only the statistically significant coefficients (Equation (3) to Equation (7)):

(3) $$Y_{DW}=408.105+17.384P-80.007S+14.13t^{2}-28.218t^{2}-4.865t^{2}P^{2}S^{2}$$

(4) $$Y_{TPC}=61.64-0.385t+2.597P-6.451S-8.872S^{2}-0.734t^{2}P^{2}S^{2}$$

(5) $$Y_{DPPH}=28.649+1.857P-3.113S+4.563t^{2}+3.026P^{2}-2.174S^{2}-1.422t^{2}P^{2}S^{2}$$

(6) $$Y_{ABTS}=170.106-43.670t+61.489P+121.825S-33.331P^{2}-28.079S^{2}+15.44\;t3-30.28P^{3}-2.174S^{3}-1.422t^{2}P^{2}S^{2}$$

(7) $$Y_{BCM}=0.095-0.005P-0.021S-0.019S^{2}+0.014P^{2}+0.009S^{2}+0.003tPS$$

In general, the cubic term had no discernible effect on the model except for ABTS, while the linear, quadratic, and interaction effects were statistically significant (*p* < 0.05) for all the parametric responses (Table 2(A)). Table 2(A) shows that the adjusted coefficient of determination (*R*^2^) for all responses ranged from 0.8608 to 0.9290, demonstrating a good fit between the experimental data and the regression models.

**Table 2:** (**A**) Parametric results of the third-order polynomial equation of Equation (1) for MAE assessed and in terms of the extraction behavior for the five evaluated responses (*yield*, *TPC*, *DPPH*, *ABTS*, and *BCM*). Variables are presented in codified ranges and the parametric subscripts 1, 2, and 3 refer to the variables involved (*X*_1_, *X*_2_, and *X*_3_). Statistical information for the fitting analysis is also shown. (**B**) Optimum conditions in natural values that lead to optimal response values formats assessed.

Coefficients		Parametric Responses to the CCCD									
		*Extract*		*Chemical*		*Antioxidant Activity*					
		*Yield*		*TPC*		*DPPH^•^*		*ABTS^•+^*		*BCM*	
***(A) FITTING COEFFICIENTS OBTAINED***											
Intercept	b_0_	408.105	±17.983	61.645	±2.237	28.649	±2.473	170.106	±14.562	0.095	±0.006
Linear effect	b_1_	ns		−0.385	±0.131	ns		−43.670	±23.646	ns	
	b_2_	17.384	±10.519	2.597	±1.309	1.857	±1.006	61.489	±23.646	−0.005	±0.003
	b_3_	−80.007	±10.519	−6.451	±1.309	−3.113	±1.006	121.825	±23.646	−0.021	±0.003
Quadratic effect	b_11_	ns		ns		4.563	±1.636	ns		ns	
	b_22_	ns		ns		3.026	±1.636	−33.331	±11.353	ns	
	b_33_	−28.218	±16.798	−8.872	±2.090	−2.174	±1.636	−28.079	±11.353	−0.019	±0.003
Cubic effect	b_111_	ns		ns		ns		15.440	±9.306	ns	
	b_22_	ns		ns		ns		−30.280	±9.306	ns	
	b_333_	ns		ns		ns		−55.446	±9.306	ns	
Interactive effect	b_12_	ns		ns		ns		ns		ns	
	b_13_	ns		ns		ns		ns		ns	
	b_23_	ns		ns		ns		14.363	±5.008	ns	
	b_123_	ns		ns		ns		ns		0.003	±0.001
	b_1122_	ns		ns		ns		ns		ns	
	b_1133_	ns		ns		ns		ns		ns	
	b_2233_	ns		ns		ns		ns		ns	
	b_112233_	−4.865	±2.022	−0.734	±0.252	−1.422	±0.332	2.760	±1.839	ns	
	*R^2^*	0.8916		0.9290		0.8792		0.8871		0.8608	
***(B) OPTIMAL CONDITIONS AND RESPONSE VALUES***											
**Optimum conditions**	*X_1_: t* (min)	14.00	±1.87	3.00	±0.87	3.00	±0.87	7.57	±1.38	25.00	±2.50
	*X_2_: P* (bar)	20.00	±2.24	20.00	±2.24	20.00	±2.24	14.25	±1.89	2.00	±0.71
	*X_3_: S* (%)	7.85	±1.40	43.49	±3.30	46.58	±3.41	71.94	±4.24	26.04	±2.55
**Responses**		mg/g dw		mg/g dw		nM R^•^/g dw		nM R^•^/g dw		µM βC/g dw	
		494.05	±54.64	67.37	±18.47	53.42	±11.72	275.94	±14.48	0.11	±1.48

*Abbreviations:* ns: non-significant coefficient; *R^2^*: correlation coefficient.

### 3.2. Influence of Extraction Conditions on Response Variables

**Figure 1:**
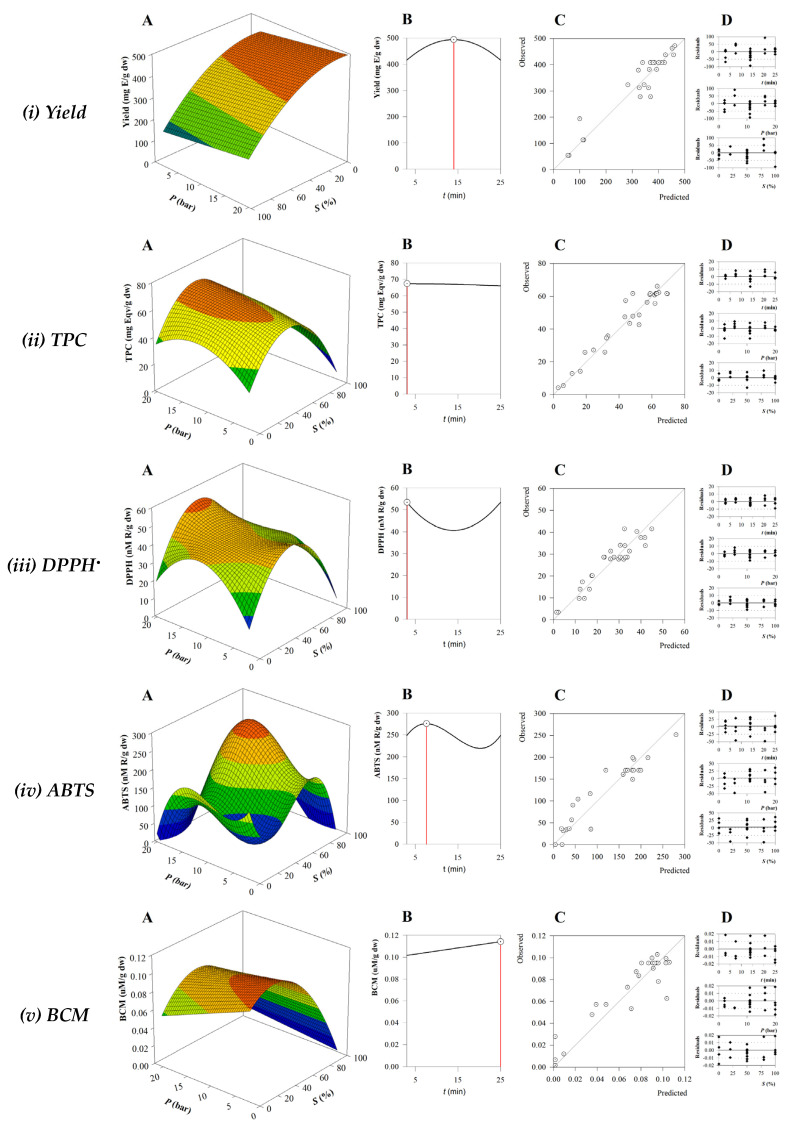
(**A**) RSM plots of the combined effect of two independent variables (*P* and *S*), with *t* at its central value; (**B**) plot of the response variable in function of *t*; (**C**) model of the predicted vs. experimental values; and (**D**) residues distribution.

The three-dimensional surface plots generated by the RSM analysis are displayed in Figure 1A. These graphs, maintaining the time variable held at an intermediate value, show the combined effects of two independent variables (*P* and *S*) on each response. The parameter values obtained from Equation (2) made it possible to obtain the response patterns mentioned above. In addition, a two-dimensional plot of the response variable vs. t is represented in Figure 1B. Figure 1C plots the predicted values against the experimental values and provides a visual representation of the correlation obtained. Finally, the distribution of residuals concerning each extraction variable is shown in Figure 1D. This distribution was always randomly distributed and showed no autocorrelation around zero. A strong correlation between the predicted and experimental values was also observed, suggesting that the generated models (Equation (3) to Equation (7)) provide insight into the phytochemical components and antioxidant activity of the BB extract.

In terms of extraction yield, the most influential element was solvent concentration, both in linear and quadratic terms (Table 2). The negative values obtained indicated that using lower ethanol concentrations led to increased extraction yields (Figure 1(i)), suggesting that most extractable chemicals such as polysaccharides and polyphenols obtained from algae are highly polar. This effect is seen in the yield results of runs 3, 5, and 7. Another factor affecting extraction yield is pressure and thus equilibrium temperature. Higher pressures lead to higher extraction yields.

Regarding the TPC, the obtained optimization model shows the relevance of the linear effect, which also includes a quadratic term related to the composition of the solvent, as well as an interactive effect (Table 2). As can be seen in Figure 1(ii), the TPC amount is favored by a higher pressure and a moderate ratio of ethanol to water. The worst TPC values were obtained when 100% ethanol was used (runs 16, 18, 20, 20, and 22). Extraction time does not seem to have a relevant effect on TPC extraction, and this behavior of TPC versus irradiation time has been previously reported [43]. In addition, studies on the optimization of phlorotannins from *Fucus vesiculosus* by MAE have shown that the extraction of this important family of phenolic compounds is favored by a medium solvent concentration (57%, ethanol), a moderate temperature of 75 °C, and a low extraction time (5 min) [44], which is consistent with our results.

When optimizing the independent variables as a function of the responses to antioxidant capacity, it was found that the models obtained were more complex and the quadratic and cubic effects became statistically significant. The variations of the optimal conditions for the different antioxidant reactions result from the diverse processes used in the antioxidant assays. Hydrogen atom transfer assays such as BCM, which deliver a hydrogen atom, differ from single-electron transfer assays such as DPPH^•^ and ABTS^•+^, which rely on the donation of an electron [45].

As for DPPH^•^, we can observe that the optimization of the scavenging extract shows a similar pattern to that of TPC with better results being at higher pressures and lower solvent ratios. A similar trend was observed in the optimization of phytochemicals from *Ascophylum nodosum* [22]. For BB extracts, Silva et al. [46] also observed a positive correlation between TPC value and antioxidant activities evaluated by DPPH^•^ scavenging activity, FRAP, and ORAC assays.

The most complex fitting equation of the optimization procedure was obtained for ABTS^•+^, response Equation (6), as both linear quadratic and cubic effects were significant (Table 2). The two independent variables, pressure and solvent, have a positive effect, while time has a counterproductive effect on the ABTS^•+^ response. The complexity of the model may reflect the complexity of the algae matrix as a variety of compounds are present in the extract and the ABTS^•+^ method is sensitive to the interaction or degree of polymerization of the phenolic compounds present [47,48]. The influence of the independent factors on the BCM results has a negative effect on the ability of β-carotene to resist oxidation, as can be seen in Table 2. This conclusion, based on the statistical significance of its linear components, indicates that lower solvent concentrations and pressures are required to prevent the degradation of this pigment. The primary factor affecting the preservation of β-carotene under the extraction conditions was the composition of the solvent, a similar behavior to a previous study [22] in which the antioxidant capacity, based on the BCM of *A. nodosum* MAE extract, was maximized when low ethanol levels and pressure were used.

Based on the RSM results, a simultaneous optimization was created to determine the maximum extraction of antioxidant-rich components from the BB extract. Our strategy was to maximize the results predicted by the existing models. As a result, it was determined that the following parameters would be optimal for the MAE: 3.39 min (*t*), pressure of 15.03 bar (*P*), and ethanol concentration of 48.53% (*S*). The expected values for each reaction under these ideal conditions were then calculated and are shown in Table 2(B). These results indicate that reaching the optimum condition to optimize a particular reaction can negatively affect other reactions. This highlights the importance of using simultaneous optimization techniques.

### 3.3. Biological Activity of the BB Optimal Extract

Once the optimal extraction conditions were established, the content of polyphenolic compounds of the optimal extract was determined. These compounds are known for their activity as antioxidants and for being linked to other bioactive properties. The value obtained for TPC was 45.6 mg Phl/g E in agreement with the results obtained in another study for a BB ethanolic extract [49]. That study mentions the presence of organic acids such as quinic acid, malic acid, and flavanone hesperidin [49]. These values are commonly found in brown alga [50]; however, they are higher than the ones reported for ultrasonic-assisted extraction, 19.9 mg Phl/g [51], and dichloromethane methanol solid/liquid extraction (1:1. *v*:*v*), reported as being 9.6 mg Phl/g [52]. Regarding the DPPH^•^ and ABTS^•+^ free-radical scavenging activity demonstrated by the BB extract, values of 57.31 nM DPPH^•^/g and 86.09 nM ABTS^•+^/g were established.

#### 3.3.1. Antimicrobial Activity

The pursuit of new molecules with the capacity to constrain the growth of pathogenic microorganisms is nowadays an important question that concerns the scientific community and the public. As reported earlier, each year contaminated food results in 600 million foodborne disease cases worldwide and 420,000 fatalities [53]. So, extracts with antimicrobial activity can help control pathogens outbreaks. In this sense, the inhibition capacity of BB extracts was studied against five foodborne pathogens, *S. aureus*, *E. coli*, *B. cereus*, *S. enteritidis*, and *P. aeruginosa*, and also bacteria related to hospital infections, *S. epidermidis.* Results are shown in Table 3.

The BB MAE extract at 20 mg/mL concentration presented activity against both Gram-positive and Gram-negative bacteria, being able to successfully inhibit the growth of all microorganisms assessed except for *E. coli.* The most expressive effect was achieved in the inhibition of *S. enteritidis* and *B. cereus* with a MIC of 1.2 mg/mL. The *S. epidermidis* growth was disturbed by an extract concentration higher than 0.4 mg/mL but the inhibition only occurred at 8 mg/mL. The determination of inhibition zones by diffusion methods, particularly when testing complex mixtures such as crude extracts, can lead to inconsistent results due to differences in the diffusion rates of various constituents, and the determination of MIC is normally considered more accurate [54].

The antimicrobial activity of BB extract was studied previously. The dichloromethane extracts corresponding to the lipidic fraction rich in sterols, namely, fucosterol, proved to be active against Gram-positive, two strains of *S. aureus* (ATCC6538 MIC = 1 mg/mL; ATCC43300 MIC = 2 mg/mL) and *S. epidermidis* (MIC > 2 mg/mL), and Gram-negative bacteria, *E. coli* 25922 (MIC = 2 mg/mL) and *P. aeruginosa* (MIC > 2 mg/mL) [55]. In another study [12], the ability of the dichloromethane and MeOH extracts of BB to inhibit *E. coli* and *S. aureus* was studied by the plate-diffusion method and the authors concluded that the tested extracts showed activity against both microorganisms. Notwithstanding this, algae extracts are commonly more effective against Gram-positive than Gram-negative bacteria, due to differences in cell wall structure [12]. Lipidic fractions of the BB extract (methanol and dichloromethane 1:4 *v*:*v*) had shown inhibition capacity towards *E.coli* [12]. These differences could be explained by the different extraction methodologies applied. The results reported on the antimicrobial activity of mid-polar (hexane–isopropanol–water (10:80:10) BB extracts demonstrated the capacity to inhibit the growth of several pathogens including *B. cereus* (MICs from 0.4–6.4 mg/mL) and *S. aureus* (MICs from 12.8–23.9 mg/mL) highlighting the seasonal and special variability of antimicrobial capacity [56].

The consistent performance of the BB extracts as an antibiotic agent could be related to the presence of diterpenes. Studies have shown the activity of these compounds against several microorganisms [57,58]; although these compounds are quite rare in nature, they are found in the *Sargassaceae* family, especially in BB species [9].

**Table 3:** Biological activities of the optimized BB extract: (**A**) in vitro antimicrobial activity against Gram-negative and Gram-positive pathogenic bacteria (by agar diffusion and microdilution methods); (**B**) inhibitory activity against reactive oxygen (ROS) and nitrogen species (RNS); (**C**) inhibition of central nervous system-related enzymes; (**D**) anti-inflammatory activity; (**E**) cytotoxic effects.

**A: Antimicrobial activity**			
	*MIC* (mg/mL)	*Inhibition zone* (mm)	
*Escherichia coli*	NI	NI	*Lactic acid 40%* (18.96 ± 3.59)
*Staphylococcus epidermidis*	8	15.86 ± 1.43	*Lactic acid 40%* (17.43 ± 3.41)
*Bacillus cereus*	1.2	5.45 ± 1.06	*Lactic acid 40%* (17.10 ± 3.36)
*Staphylococcus aureus*	>8	12.06 ± 1.88	*Lactic acid 40%* (19.08 ± 4.59)
*Salmonella enteritidis*	1.2	9.77 ± 1.63	*Lactic acid 40%* (18.06 ± 2.93)
*Pseudomonas aeruginosa*	>8	10.86 ± 0.23	*Lactic acid 40%* (19.45 ± 1.45)
**B:** **Antioxidant activity (ROS, RNS)**			
	*IC*_50_ (µg/mL)		*IC*_50_ (µg/mL)
Hydroxyl radical (^●^OH)	446.1 ± 22.3		*Ascorbic acid* (183 µg/mL)
Nitric oxide (^●^NO)	55.35 ±2.76		*Ascorbic acid* (446 µg/mL)
Superoxide anion (O_2_^●–^)	44.25 ± 2.21		*Ascorbic acid* (160 µg/mL)
Hydrogen peroxide (H_2_O_2_)	108.4 ± 5.4		*Ascorbic acid* (51 µg/mL)
**C:** **Enzymatic inhibition**			
	*IC*_50_ (µg/mL)		*IC*_50_ (µg/mL)
Tyrosinase	329.6 ± 16.4		*Kojic acid* (1.82 µg/mL)
Monoamine oxidase A (MAO-A)	133.6 ± 6.68		*Clorgyline* (25 ng/mL)
Monoamine oxidase B (MAO-B)	154.8 ± 7.74		*Selegiline* (2.1 µg/mL)
Acetylcholinesterase (AChE)	>2000		*Galantamine* (0.92 µg/mL)
Butyrylcholinesterase (BuChE)	712.9 ± 35		*Galantamine* (4.92 µg/mL)
**D: Anti-inflammatory activity**			
	*IC*_50_ (µg/mL)		*IC*_50_ (µg/mL)
RAW264.7	320.5 ± 10.6		*Dexamethasone* 7.23 ± 0.85
**E: Cytotoxic effects**			
	*IC*_50_ (µg/mL)		*IC*_50_ (µg/mL)
A549 (lung adenocarcinoma)	87.57 ± 2.04		*Ellipticine* (<1 mg/mL)
HEPG2 (hepatocellular carcinoma)	52.85 ± 2.71		*Ellipticine* (0.85 ± 0.046)
AGS (gastric adenocarcinoma)	38.75 ± 2.18		*Ellipticine* (<1 mg/mL)
Vero	85.85 ± 2.67		*Ellipticine* (<1 mg/mL)

Abbreviations: MIC, minimum inhibitory concentration; Vero, the African green monkey kidney-derived cell line; AGS, the human gastric cancer cell line; A549, the human lung adenocarcinoma cell line; HEPG2, the human hepatocarcinoma cell line.

#### 3.3.2. Reactive Oxygen and Nitrogen Scavenging Activity

**Figure 2:**
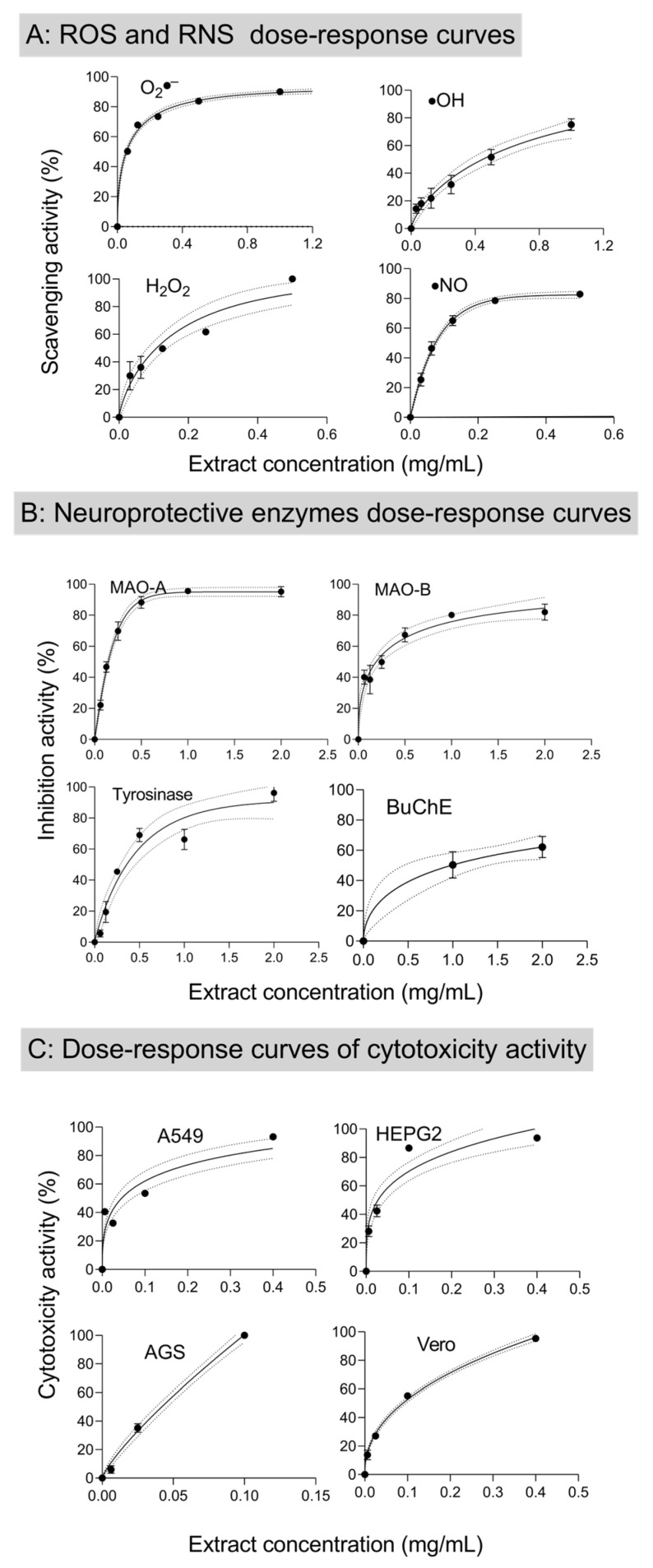
Weibull model fitting curve of dose–response relationship of BB scavenging activity to: (**A**) ROS and NOS (O_2_^•−^, H_2_O_2_,∙^•^NO and ^•^OH); (**B**) inhibition activity of MAO–A, MAO–B, BuChE, and tyrosinase enzymes; and (**C**) anti-proliferation of HEPG2, AGS, A459, and Vero cells. Dotted bands represent 95% confidence levels. Error bars represent the standard deviation.

DNA damage is one of the recognized causes of health degradation related to life-threatening diseases, namely, cancer. Reactive oxygen and nitrogen species such as hydrogen peroxide (H_2_O_2_), superoxide anion (O_2_^●−^), hydroxyl radicals (^•^OH), and nitric oxide (^•^NO), known causes of oxidative stress, participate in the inflammatory process and are related to DNA damage [59,60]. Moreover, the reactive oxygen species are also a major cause of food spoilage by the destruction of biomolecules and causing rancidity [61]. The BB extract capacity to scavenge the ROS and RNS was evaluated and the dose–response curves are presented in Figure 2. The results obtained are efficaciously represented by the Weibull model, with a coefficient of determination higher than 0.9, allowing the calculation of the IC_50_ of BB MAE ethanolic extract scavenging activity (Table 3).

Previous studies have reported that brown alga extracts have ^•^NO scavenging capacity [35,62,63]; similarly, BB extracts were capable of successfully interacting with the ^•^NO, presenting an IC_50_ of 55.35 ± 2.76 µg/mL, significantly lower than the positive control (ascorbic acid, IC_50_ = 446 µg/mL). This strong activity is in line with a previous study [64] reporting that a hexane/ethanol (1:1, *v*/*v*) extract of BB with a TPC value of 2.60 μg phloroglucinol equivalents/mg residue, total carbohydrate content of 5.84 μg glucose equivalents/mg residue, and citric and α-hydroxybutyric acids as major components, was able to counteract ^•^NO production in Caco2 cells treated with tert-BOOH. Moreover, carotenoids, such as fucoxanthin, can suppress the expression and secretion of inflammatory mediators such as nitric oxide [65].

The alga extracts also presented an important O_2_^•−^ scavenging activity, displaying an IC_50_ of 44.25 ± 2.21 µg/mL. Regarding O_2_^•−^ scavenging activity, there are no comparable data in the published literature, but one work [61] is available reporting the superoxide scavenging capacity of seven species of brown alga. Among these data, the best-performing algae were *E. cava* Termamyl (enzymatic extract) which presented a superoxide anion scavenging activity of ≈68% for a concentration of 2 mg/mL [61]. *E. cava* extracts were those presenting the highest TPC values [61]. Nonetheless, not only phenolic compounds are responsible for the O_2_^•−^ scavenging activity, also carotenoids are strong O_2_^•−^ scavengers [66,67].

The IC_50_ attained towards the hydroxyl radical was 446.1 ± 22.3 µg/mL and the BB extract was also an interesting scavenger of the H_2_O_2_ molecule with an IC_50_ of 108.4 ± 5.4 µg/mL. These characteristics are very important since a study in renal epithelial cells showed that scavengers of hydrogen peroxide inhibited necrotic cell death, whereas scavengers of hydroxyl radicals prevented apoptosis by inhibition of cytochrome c release and caspase activation [68]. It is also worth highlighting that the performance of BB crude extract as ROS and NOS scavenger was better than the reference substances for the species O_2_^●−^ and ^•^NO. The scavenging properties of BB extract are biologically relevant since the ^•^NO and the O_2_^•−^ radicals are the precursors of other free radicals, such as peroxynitrite, which can have adverse effects since it is a strong oxidizer and contributes to various diseases related to oxidative stress [69].

#### 3.3.3. In Vitro Neuroprotective Properties

Progressive neurological disorders are a major problem affecting the world population, with special emphasis on the elderly. Alzheimer’s and Parkinson’s diseases have an enormous impact on the quality of life of patients and thus on the importance of therapies to improve the associated symptoms. Furthermore, these brain disorders have been also associated with clinical depression [70].

Cholinesterase inhibitors act by preventing the hydrolysis of acetylcholine in the synaptic gap of neurons. The collapse of this neurotransmitter, related to cognitive function, leads to cholinergic dysfunction and, in the end, to memory loss [35]. One of the major available strategies for Alzheimer’s disease treatments is the inhibition of AChE [71]. The role played by BuChE inhibitors is also critical being an important therapeutic target in Alzheimer’s therapy [72]. The BB extract was evaluated as a potential neuroprotective agent and at the highest concentration tested (2 mg/mL) led to an AChE inhibition rate of 14.2 ± 2.0%. The extract was a better BuChE inhibitor, inhibiting 61.6 ± 6.4% of the enzyme at 2 mg/mL (Figure 2). The presence of phenolic compounds and carotenoids in BB extract may be responsible for this inhibition since these classes of compounds have shown remarkable activity towards these enzymes [73,74,75].

Previous work on the enzyme inhibition capacity of BB reported IC_50_ of 141 µg/mL against AChE and IC_50_ of 177 µg/mL against BuChE in the extract obtained by maceration (70% ethanol, 60 °C, 2 h), whose main detected compound was quinic acid. Although these results are not directly comparable due to differences in the sampling and sample preparation, they highlight the potential of BB extracts as neuroprotective agents [49]. Moreover, the neuroprotective effect of BB dichloromethane extract fractions (Vacuum Liquid Chromatography, cyclohexane/ethyl acetate) was evaluated in SH-SY5Y treated with the neurotoxin 6-hydroxydopamine (6-OHDA), corresponding to an in vitro model of Parkinson’s disease. The results demonstrated the neuroprotective effect, linked to the reduction of oxidative stress via decreasing H_2_O_2_ production, anti-apoptotic effect, and Caspase-3 inhibition [10]. In a continuation of that study, the authors [46] tested several methanolic and ethyl acetate fractions isolated from BB in the same model, showing that the fraction containing mannitol displayed the strongest neuroprotective effect by increasing cellular viability and decreasing ROS production and caspase-3 activity.

Studies on three molecules isolated from BB, eleganolone, eleganonal, and fucosterol, found that elonganolone corrected the neurotoxicity caused by 6-OHDA by roughly 20%. The neuroprotective benefits were mediated by mitochondrial protection, oxidative stress reduction, inflammation and apoptosis decrease, and suppression of the NF-kB pathway [76].

Another relevant therapeutic option is monoamine oxidase inhibitors, which are effective in Alzheimer’s and Parkinson’s diseases. MAO is a flavoenzyme with two isoforms (MAO–A and MAO–B) that engage in the oxidative deamination of monoamines. The MAO–A substrates are noradrenaline and serotonin, while in MAO–B it is dopamine. MAO inhibitors also have effects as antidepressants [77].

Regarding the MAO–A and -B inhibition, the achieved results are more expressive since 1 mg/mL was able to promote a 74.4 ± 8.4% inhibition rate for MAO–A and 85.9 ± 0.85% for MAO–B (Figure 2). The high inhibition rates detected allowed us to study the dose–response behavior and calculate the IC_50_ values of 133.6 ± 6.68 and 154.8 ± 7.74 µg/mL, respectively. As far as we know, there is no previous report on the MAO inhibition capacity of BB. However, there is a previous work supporting the interaction between brown algae polyphenols and both MAO isoforms [78]. Indeed, the methanolic extract of *E. bicyclis* and the phlorotannins eckol and dieckol were tested against both isoforms, revealing strong activities [79]. On the other hand, other classes of compounds also showed promising effects, such as monocyclic terpenoid lactones [80] and carotenoids [81].

Tyrosinase is a key enzyme in melanin biosynthesis via the production of L-DOPA. This enzyme also contributes to neuromelanin formation. The neuromelanin-containing neurons deteriorate selectively in Parkinson’s disease [25]. Tyrosinase inhibition accomplished in the presence of the optimized BB extract was also remarkable. An extract concentration of 1 mg/L led to 66.2 ± 6.5% inhibition and the IC_50_ determined was 329.6 ± 16.4 µg/mL. The tyrosinase inhibition capacity of a 70% ethanolic extract of this alga (maceration) was reported as having an IC_50_ > 0.20 mg/L; even though the extraction technique is different, both results agree well [49]. The strong activity observed for BB extracts may be correlated with the presence of phlorotannins, known for their capacity to inhibit this enzyme [82].

#### 3.3.4. Anti-Inflammatory and Cytotoxic Properties

To study the anti-inflammatory capacity of BB, an in vitro experiment based on the •NO production by macrophages stimulated with LPS was performed. The results obtained show that the BB extract has anti-inflammatory activity, presenting a GI_50_ = 320.5 µg/mL. The anti-inflammatory capacity of the fucosterol-rich lipidic fraction of BB was already studied, revealing a significant anti-inflammatory potential of this dichloromethane-based extract [55]; in that study, a 50 µg/mL extract inhibited LPS-induced NO production to 6%. That result unveiled the anti-inflammatory potential of BB.

The cytotoxicity of the BB extract was tested in several human cancer cell lines and non-tumor cell cultures; the summary of the results is shown in Figure 2 and the respective GI_50_ in Table 3. Although the results obtained in the A549 cell line (human adenocarcinoma) were within the boundary of the hepatotoxicity, they were very encouraging for the two other cell lines (AGS, HepG2), featuring the best results in the detaining of the growth of gastric adenocarcinoma cell and hepatocellular carcinoma with low GI_50_. Previous studies showed that dichloromethane and methanol extracts of BB affect the proliferation of human colorectal adenocarcinoma and human hepatocellular liver cancer [9,83], a feature also shown by the (hexane/ethanol, 1:1, *v*/*v*) BB extract that had an anti-apoptotic effect by increasing the Caco-2 cells resistance against oxidative stress, and by inhibiting the caspase 3/7 activity [64].

In the literature, the anticarcinogenic effect on the bronchopulmonary carcinoma cell line has been attributed to a possible inhibition impact in the G1 phase (when the cell synthesizes mRNA and proteins) of the cell cycle [27]. A different study revealed the activity of dichloromethane methanol (1:1, *v*:*v*) BB extract, obtained by accelerated solvent extraction, against human cancer cell lines of lymphoma (Daudi), leukemia (Jurkat), and erythroleukemia (K562); data showed the 100 μg/mL crude extract inhibits all the cell lines at least 60% [52].

## 4. Conclusions

The optimization of microwave-assisted extraction (MAE) of the macroalga *Bifurcaria bifurcata* aimed to produce an antioxidant-rich extract, and it led to the establishment of the optimal conditions as an irradiation time of 3.39 min, a pressure of 15.03 bar, and an ethanol concentration of 48.53%. The resulting extract demonstrated effective antimicrobial activity by inhibiting the growth of all tested bacteria, except *E. coli*. Moreover, the BB extract showed potential in oxidative stress prevention by acting as a scavenger of reactive oxygen species and reactive nitrogen species.

Regarding its neuroprotective properties, although the extract exhibited weak activity as an AChE inhibitor, it showed a stronger potential as a BuChE inhibitor. Additionally, it is noteworthy that the BB extract inhibited the enzymes MAO–A, MAO–B, and tyrosinase, all of which are associated with neurodegenerative diseases. The extract also demonstrated anti-inflammatory properties, and cytotoxic effects, reducing cell growth in lung adenocarcinoma, hepatocellular carcinoma, and gastric adenocarcinoma. In the future, a comprehensive analysis of the BB extract is necessary to identify the specific chemical compounds responsible for these significant bioactive properties.

## Data Availability

Data are contained within the article.
